# Patient Engagement in a Hybrid Care Pathway for Hypertension: Not One Size Fits All

**DOI:** 10.1177/23743735241297626

**Published:** 2024-12-08

**Authors:** Job van Steenkiste, Iris Verberk-Jonkers, Stéphanie de Koning, Joyce Voss-de Haan, Bianca de Jong-Verhagen, Daan Dohmen

**Affiliations:** 1 Faculty of Management Sciences, 10198 Open University, Heerlen, the Netherlands; 2 Department of Internal Medicine, 7000 Maasstad Hospital, Rotterdam, the Netherlands; 3 Department of Hospital Pharmacy, 6993 Erasmus MC University Medical Center, Rotterdam, the Netherlands

**Keywords:** hybrid care pathways, hypertension, digital care, digital lifestyle intervention, patient engagement

## Abstract

We evaluated current experiences and future needs for the long-term engagement of patients in a hypertension hybrid care pathway (Maasstad Hospital, NL). Patients >18 y/o with ≥3 months care pathway participation were recruited by telephone and divided into three age/focus groups with distinct digital skills and attitudes toward lifestyle interventions (group 1:18-40 y/o, group 2:40-65 y/o, group 3:>65 y/o). We used deductive thematic content analysis to cluster the results to the different digital elements (remote monitoring, communication, digital lifestyle intervention) of the care pathway. Fifteen patients were interviewed in March 2023 (Group 1;2;3, 3;6;6 participants). The care pathway improved disease insight, engagement, and shared decision capabilities in all groups. For further improved adherence and engagement, all patients indicated the need to incorporate a more personalized approach and interaction with their healthcare provider. In addition, the oldest group preferred simplification of the telemonitoring application, while the other groups preferred enrichment with more complex information. To ensure optimal engagement, digital elements in hybrid care pathways should be personalized and tailored to individual as well as to age-specific needs.

## Key Findings

– Patients included in a hybrid care pathway for hypertension reported improved disease insight, engagement, and shared decision capabilities.
– Patients included in a hybrid care pathway for hypertension indicated that the digital elements of the pathway should be more personalized and tailored to individual needs.
– Patients included in a hybrid care pathway express an ongoing need to have personal interactions with their own healthcare providers to improve adherence and engagement.
– Differences exist between age groups with regard to the content and functionalities of the telemonitoring application used in the care pathway, where the oldest group preferred simplification of the app, while the other groups preferred enrichment with more complex information.

## Introduction

Hypertension is a major public health challenge, and its global prevalence is estimated to increase by 15% to 20% to a total of 1.5 billion affected people by 2025.^1^ Despite lifestyle interventions and drug therapy, many patients with hypertension remain off-target, partly due to non-adherence.^2^ In addition to the clinical challenges, there has recently been an increasing organizational challenge in these patients.^3^ An increased care demand combined with decreased healthcare staffing requires different care organizations. To tackle both the clinical and organizational challenges, the use of hybrid care pathways has been proposed. In a hybrid care pathway, patients receive a mix of digital and physical care which allows for efficient care delivery as well as improved adherence, disease insight, and patient engagement.^4^ The digital part of these care pathways consists of digital communication tools, home blood pressure telemonitoring (HBPT), and digital lifestyle interventions. Physical care is standard care provided via the outpatient clinic.

### Digital Communication

Digital communication tools are important elements as they allow direct and indirect communication between patients and healthcare providers. This ensures adherence and engagement to drug therapy or lifestyle interventions which can ultimately lead to better blood pressure control, reduced adverse events, and improved clinical outcomes.^4^ Also, digital communication between patients and healthcare providers is generally considered to be more efficient and less time-consuming.^5^

### Home Blood Pressure Telemonitoring

With HBPT patients measure their blood pressure at home while being remotely monitored by their healthcare providers. HBPT has significantly evolved over the last years from protocolized self-monitoring to proactive telemonitoring using telemonitoring applications that generate clinical alerts in off-target patients.^6^ This provides early detection, allowing healthcare providers to optimize treatment if needed.^6^ Additionally, on-target patients require less contact compared to usual care, as the absence of alarms reassures that the target blood pressure is met. Recent meta-analyses on the clinical effectiveness of HBPT showed significant improvements in blood pressure control, but specific data on proactive telemonitoring are missing.^7^ In addition, many hospitals now offer specialized monitoring centers where e-nurses monitor patients with hypertension for example. These centers have been developed to organize care as efficiently as possible as well as to improve clinical outcomes.

### Digital Lifestyle Intervention

Modern-day HBPT platforms can also incorporate digital lifestyle interventions. These include educational programs to improve disease insight and therapy adherence, which can be delivered proactively. Some randomized trials have specifically demonstrated a positive effect of HBPT on lifestyle factors (eg reduced sodium intake), however, the HBPT programs used in these trials did not incorporate a digital lifestyle intervention.^8^ On the other hand, trials evaluating specific digital lifestyle interventions did not incorporate HBPT.^9^ Combining pro-active HBPT with digital lifestyle interventions, however, can possibly lead to enhanced antihypertensive effects.^10^

Integrated scientific evaluations that address all hybrid care pathway elements are missing. This is especially relevant for chronic diseases as ongoing engagement is required for improved long-term clinical outcomes. In addition, it is known that different ages and generations have different digital skills and attitudes toward lifestyle interventions for chronic diseases.^11,12^ To provide more guidance on the development of a hybrid care pathway we conducted a qualitative analysis using three focus groups (18-40 y/o, 40-65 y/o, >65 y/o) of experienced (>3 months) patients included in a hybrid care pathway for hypertension in the Maasstad Hospital in Rotterdam, the Netherlands. We evaluated patients’ current experiences and future needs and extracted important elements to be considered to ensure long-term engagement.

## Methods

This study was conducted in the Maasstad Hospital, a 600-bed teaching hospital in Rotterdam, the Netherlands.

### Hybrid Care Pathway for Hypertension

**Figure 1.**
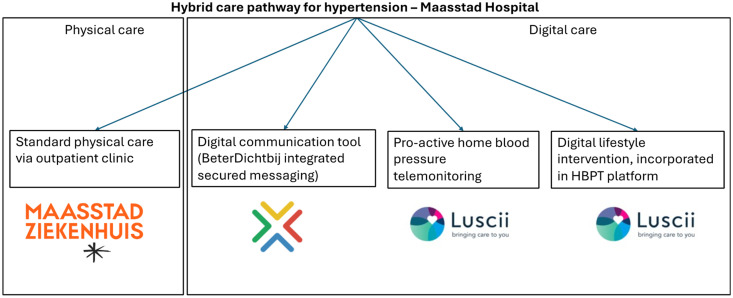
Four different elements of the hybrid care pathway for hypertension evaluated in this study.

The specific hybrid care pathway (Figure 1) for hypertension evaluated in this study consists of all four different elements described in the introduction: physical care, a proactive HBPT program, a digital lifestyle intervention, and a digital communication tool. Physical care is usual outpatient department care which includes physical consultations and blood pressure measurements.

Patients receive instructions from their healthcare providers in terms of the functionalities of the app and the organization of the remote monitoring service. The patient information department provides all the necessary technical instructions. If the patient experiences physical or technical issues, he, or she can contact the hospital during office hours.

### Digital Communication

Communication takes place between the patient and the outpatient clinic, the monitoring center, or the healthcare provider. This is done by telephone or via an integrated (asynchronous) messaging platform.^13^ This platform can be accessed from the HBPT application. From the hospital's side, the messaging platform is integrated into the electronic health record (EHR), and standardized messages are used to improve efficiency. Digital communication takes place between the monitoring center and the patients in the event of abnormal blood pressure values (monitoring center) or following a period with on-target blood pressure values (standardized messages via the HBPT application). During all communication interactions, both with the monitoring center and with the healthcare provider, attention is paid to the relevant blood pressure values (insight into abnormal values) and the importance of lifestyle interventions.

### Remote Monitoring Program and Telemonitoring Procedures

The HBPT program has been built in the Luscii telemonitoring application.^14^ It includes a predefined measurement schedule that is dependent on patients’ individual blood pressure targets. Patients measure their blood pressure based on their level of blood pressure control (daily, one week every fortnight, or one week every month). They measure twice in the morning and twice in the evening as recommended by the guidelines.^15^ Patients are reminded to perform these measurements via push notifications. The measurements are transmitted using a validated Bluetooth^®^ blood pressure machine to the app. The program includes threshold values that trigger alerts in off-target patients.

The telemonitoring itself is conducted by a monitoring center in the Maasstad Hospital. Here, specialized e-nurses review the alerts and measurements on a daily (Monday to Friday) basis. They are the first line of contact with the patients and are supervised daily by clinical nurse specialists or doctors. Interactions between the patients and the monitoring center occur based on the alerts for off-target (high and low) blood pressure values. The e-nurses would always try to find out whether the patient adheres to the relevant lifestyle factors or drug therapy and can provide additional support or direct the patients toward the lifestyle intervention in the app.

### The Digital Lifestyle Intervention

The digital lifestyle intervention in the HBPT app consists of educational lessons and information files on relevant topics (see Supplementary File 1 for the complete overview and examples). All material is accessible at any time, is developed at the B1 language level to ensure accessibility, and is largely based on material from the Dutch Heart- and Kidney Foundations.^16,17^ Also, during each measurement week, patients are actively prompted to read through the material. Additionally, the app will automatically calculate body mass index (BMI) and has an integrated pedometer. These blood pressure data can be visualized using graphs with an adjustable timeframe.

### Participant Selection, Recruitment, and Informed Consent

We included adults (≥18 years old) who spoke Dutch and had participated in the hybrid care pathway for at least 3 months to ensure enough exposure. The only exclusion criterion was the inability to provide informed consent. Based on in- and exclusion criteria, we generated a list with ordinal numbers with current patients included in the care pathway. Each patient had a random position on the list (eg no alphabetic filter was applied). We divided this list into three age groups (1: 18-40 y/o, 2: 40-65 y/o, 3: > 65 y/o), as digital skills vary significantly between these groups.^11^ We aimed to include at least seven patients in each group to ensure data saturation and prevent overcrowded focus groups. Patients were invited consecutively from the numbered list via a telephone conversation with the patient information department. Once seven patients in each age group had agreed to participate no further patients were invited. Written informed consent was obtained from all participants before the focus groups.

### Focus Group Procedures and Data Collection

Following recruitment in February 2023, three separate focus groups based on the age groups were scheduled to be conducted in the Maasstad Hospital in March 2023. Only a notetaker, facilitator, and moderator were present during the focus groups, and they had no direct or indirect relations with the participants. All focus groups lasted for two hours and were recorded. An interview guide was used (see supplementary file 2). At the start of the focus groups, patients were asked to fill in the validated Telehealth Usability Questionnaire (TUQ)^18^ and Mhealth Usability Application Questionnaire (MAUQ).^19^ These questionnaires were used to validate the responses provided during the focus groups.

### Data Analysis

All three audio files were transcribed and analyzed independently by BV and JV. Coding was subsequently performed in Microsoft Word^20^ in a deductive way using thematic content analysis and was based on the following predefined themes:

1. Current experiences with the hybrid care pathway in achieving lifestyle goals and blood pressure targets.
2. Perceived added value of a hybrid care pathway for hypertension.
3. Optimal method of delivering support by the hybrid care pathway hospital for achieving lifestyle goals and blood pressure targets.
4. Future needs to ensure long-term engagement and durability in achieving relevant lifestyle goals and blood pressure targets.

Given the absence of existing frameworks for qualitative evaluations of hybrid care pathways, we chose these themes based on a discussion with all authors. The identified codes from the interview transcripts were discussed in a meeting with BV, JV, and IV which led to the final focus group results. These results were combined into “current experiences and future needs” and attributed to the three “digital elements” of the hybrid care pathway and the three different groups to provide a clear and structured view of the group perspectives across each digital element. No participant feedback was provided on the focus group results.

## Results

### Baseline Focus Group Characteristics

All three focus groups were conducted in March 2023. Overall, 107 patients met the predefined inclusion criteria (see supplementary file 3). Twenty-one patients were invited, and fifteen patients participated. Group 1; 18-40 y/o consisted of three participants (average age 34, 2 females), group 2; 41-65 y/o consisted of six participants (average age 49, 4 females) and group 3; >65 y/o also consisted of six participants (average age 74, 3 females). All participants had at least 4 (range 4-21) months of experience with the hybrid care pathway.

### Focus Group Results

All three groups reported improved disease insight, engagement, and shared decision capabilities following their participation in the hybrid care pathway. However, all three groups reported a clear need for a more personal approach within the care pathway. They reported a need for more personal feedback on both on- and off-target measurements, more personalized treatment goals, and more personal instructions. They also requested more personal involvement from their own healthcare providers, for example, by means of more positive and personal reinforcement when lifestyle goals are achieved.

### Digital Communication

Patients appreciated the accessible communication possibilities, but there was a clear preference throughout all age groups to receive more personal communication and have more personal interactions, preferably with their own healthcare providers. See Table 1 for a complete overview of the current experiences and future needs with regards to the digital communication part of the hybrid care pathway.

**Table 1.** Current Experiences and Future Needs Among Three Different Age Groups for the Digital Communication Part of the Hybrid Care Pathway.

	Group 1 (18-40 y/o)	Group 2 (40-65 y/o)	Group 3 (65+ y/o)
Current experiences and future needs	We need more insight into the organization behind the HBPT program (eg who processes our measurements) We need more personal feedback on both on- and off-target measurements (both positive and negative) We need more personal feedback and instructions that align with each patient’s specific needs and disease stage. We need more active push notifications that stimulate a healthy lifestyle. We need more involvement from our own healthcare providers.	We do not need a pedantic approach to relevant lifestyle interventions from our healthcare providers. We prefer physical consultations of digital or telephone consultations. We prefer to be approached by our own healthcare provider. We need more active push notifications that stimulate a healthy lifestyle.	We sometimes experience the messages provided by the app as contradictory (eg within 24 h both positive and negative messages on blood pressure measurements). We need more insight into the organization behind the HBPT program (eg who processes our measurements) We need one central point of contact. We would like to have more personal contact with our own healthcare providers. We would like to have more personal contact for off-target measurements.

### Home Blood Pressure Telemonitoring

All groups reported HBPT to have a positive impact on their self-management abilities. They also expressed the measurement functionalities of the app to be convenient to work with. On the other hand, groups 1 and 3 reported the need for more specific instructions on the functionalities of the HBPT app. Groups 2 and 3 also reported the need for more flexibility with regard to the measurement schedule in the app. They further wanted more personalized treatment goals and thresholds implemented in the app. Group 3 indicated that the app should align with an older target population and that it should remain with its primary function as a measurement transmitting platform. See Table 2 for a complete overview of the current experiences and future needs with regards to the HBPT part of the hybrid care pathway.

**Table 2.** Current Experiences and Future Needs Among Three Different Age Groups for the Home Blood Pressure Telemonitoring Part of the Hybrid Care Pathway.

	Group 1 (18-40 y/o)	Group 2 (40-65 y/o)	Group 3 (65+ y/o)
Current experiences and future needs	The telemonitoring app has a stimulating effect on self-management. We need very clear instructions at the start of the program in terms of the available functionalities in the app. We need more insight into the organization behind the HBPT program (eg who processes our measurements) It's important to have reliable measurement devices and an easy-to-use app.	We experience to have a greater sense of direction and control by performing self-measurements. The app is useful for clustering relevant information. The app makes it possible to read relevant information at a time that is convenient for each individual patient. The blood pressure data generated in the app allows for more self-management which helps in shared decision-making with. We would like to have more flexibility and a more personal approach with regard to the blood pressure measurement schedule and the provision of relevant lifestyle information We would like our GPs and direct environment also to have access to the app.	We only see the app as an instrument to transmit blood pressure measurements. We perceive the app as impersonal, which also causes us to lose confidence in our own healthcare providers. We need more insight into the organization behind the HBPT program (eg who processes our measurements) We need more personalized treatment goals, personal thresholds, and personal instructions (eg why is a certain drug added specifically to my regimen) We would like to have more flexibility and a more personal approach with regard to the blood pressure measurement schedule Besides the measurement functionalities, we do not need any additional functionalities in the app. We need more personal instructions with regard to the functionalities of the app The app should also align with an older target population. It's important to have reliable measurement devices. We want to have the possibility to provide notes in the app with each measurement. The app should allow to provide insight in order relevant values (eg cholesterol)

### Digital Lifestyle Intervention

All groups were positive about the flexible accessibility of the digital lifestyle intervention provided within the app. However, patients from groups 1 and 2 experienced the information provided in the app as superficial and requested the provision of more complex and practical information (eg pathophysiology of hypertension, healthy recipes) in the app. Group 3 reported no clear need for any additional lifestyle functionalities in the app. They would, however, appreciate the integration with other relevant health parameters (eg cholesterol) in the app. See Table 3 for a complete overview of the current experiences and future needs with regards to the digital lifestyle intervention part of the hybrid care pathway.

**Table 3.** Current Experiences and Future Needs among Three Different age Groups for the Digital Life Style Intervention Included in the Hybrid Care Pathway.

	Group 1 (18-40 y/o)	Group 2 (40-65 y/o)	Group 3 (65 + y/o)
Current experiences and future needs	The information on lifestyle therapy is experienced as impersonal and superficial. The quality of the information provided in the app is worse compared to other existing platforms like thuisarts.nl or Google. Not every patient was aware of the existence of the lifestyle program in the app. Lifestyle interventions via the app provide a good incentive to work on specific behavior. We would like to have access to an external motivator (eg doctor of dietician) to achieve a healthy lifestyle, ideally integrated in the app. We need more complex information, for example on the mechanisms of anti-hypertensive drugs, in the app. We need more active push notifications that stimulate a healthy lifestyle.. We need more practical examples, for example, recipes for healthy meals or healthy products. These examples should be delivered in an active way. We need more direct integrations with other relevant lifestyle apps.	The app is useful for clustering relevant information. The app makes is possible to read relevant information at a time that is convenient for each individual patient. The information provided in the app on lifestyle is too simple. We do not need a pedantic approach on relevant lifestyle interventions from our healthcare providers. We would like to get in touch with fellow hypertension patients to exchange ideas and experiences. We prefer to receive more guidance from the hospital with regards to nutrition We would like to have more flexibility and a more personal approach with regards to the blood pressure measurement schedule and the provision of relevant lifestyle information We need more complex information, for example on the mechanisms of anti-hypertensive drugs, in the app. We need more active push notifications that stimulate a healthy lifestyle. We need more expert guidance on lifestyle factors. We would like to be stimulated actively in achieving lifestyle goals, for example with games, rewards, or a points system. We need more practical examples, for example, recipes for healthy meals or healthy products. These examples should be delivered in an active way. We need more direct integrations with other relevant lifestyle apps. We need more support from a dietician (for example during a joint information session)	Besides the measurement functionalities, we do not need any additional functionalities in the app. We do not need additional lifestyle support via the app. The app should allow to provide insight in order relevant values (eg cholesterol)

### Questionnaires

Questionnaires were completed by 100% of the participants. Median TUQ scores were 4 [IQR 4-4], 6 [IQR 5-6], and 5 [IQR 4-6] on a 1-7 Likert scale for groups 1, 2, and 3, respectively. Median MAUQ scores were 4 [IQR 3-4], 6 [IQR 5-6], and 6 [IQR 5-6] on a 1-7 Likert scale for groups 1, 2, and 3, respectively. The results confirm sufficient satisfaction with the current care pathway as expressed in the focus groups, and the relatively lower scores on the MAUQ questionnaire of group 1 are in line with their wish for more functionalities, integrations, and complex information provided in the app.

## Discussion

Patients with hypertension included in this qualitative study reported the hybrid care pathway to contribute to improved adherence, engagement, disease insight, and shared decision-making. However, to ensure and improve long-term engagement, the individual elements of the care pathway should include some level of personalization. Both in the communication, the HBPT as well as the digital lifestyle intervention there remains a need for personal and tailored care delivery. Additionally, digital interactions should preferably be performed as much as possible by a familiar healthcare provider. According to our results, this approach improves patients to feel supported by and engaged in the hybrid care pathway which could ultimately facilitate long-term adherence and improved clinical outcomes. The element of “personalization” is important to consider when optimizing care delivery in terms of efficiency which can result in more “impersonal” and “one size fits all” approaches, for example by using standardized digital messaging. We also found differences between the various age groups, with younger patients requiring more complex information and functionalities in the digital lifestyle intervention and HBPT platform, while older patients requiring the HBPT platform to solely serve as a device to transmit blood pressure values. These findings again highlight the need for the development of various tailored and personalized elements in a hybrid care pathway, especially across different age groupsThere are no similar evaluations of patient experiences with a hybrid care pathway that includes multiple digital elements available. However, some of our specific findings, like the reported improvement of disease insight and adherence are in line with a study by McHale et al which qualitatively evaluated remote hypertension care delivery in a primary care setting during the COVID-19 pandemic.^21^ Additionally, our results are in line with a recent study by Eze et al^22^ which identified specific patient-reported barriers and facilitators for blood pressure telemonitoring. Similar to our study, blood pressure telemonitoring was found to be a facilitator for patients to improve disease insight, engagement, and self-management.

### From Theory to Practice

Establishing that the various elements of a hybrid care pathway should be personal and tailor-made for each individual patient while improving efficient care delivery poses significant challenges. HBPT platforms can in themselves be experienced as impersonal and robotic, and it can be difficult to adjust the platform to the patients’ individual level of health literacy, digital skills, and disease stages as monitoring programs are often developed as general programs for large groups of patients.^23^ However, there are some existing possibilities to overcome this. First, educational modules, measurements, and messages can be scheduled flexibly on an individual patient level. This creates a sense of personalization, which was also suggested in the focus groups, while still working with standardized instruments that improve efficiency. Second, interactions with digital messages can be made more personal by using generative large language models (LLM), for example by creating variety in the content of the message.^24^ These interactions between patients and individual healthcare providers are vitally important, both in motivating the initiation of lifestyle interventions and in ensuring long-term adherence as confirmed across all our focus groups where patients described to see their own healthcare providers as motivators. Using LLM's can therefore be particularly relevant in a setting where personal interaction with the healthcare provider is limited due to time constraints. Finally, further integration of the various digital elements, for example with the patients’ individual EHR, allows relevant information to be more targeted to the individual patient resulting in a more personalized approach. To further put our findings into practice and optimize long-term engagement, we suggest future research to focus on the development, implementation, and evaluation of more personalized hybrid care pathways that meet the primary requirement of the integration and combination of digital communication, remote monitoring, and digital lifestyle coaching.

## Limitations

There are certain limitations with regard to study size and design that need to be considered when interpreting the study results. First, data saturation during the focus groups was not completely reached due to the participation of 15 of the originally intended 21 patients. This was most evident in group 1. Second, there remains an element of selection bias, as only engaged (positive or critical) patients are likely to have taken part in the focus groups. Our study results are therefore limited in generalizability, especially for less involved patients and we realize that this specific patient group could particularly benefit from a hybrid care pathway. It therefore would have been of added value to have included patients with a lower level of engagement, such as patients included in the care pathway for less than 3 months. Third, we chose to divide the focus groups into three separate age groups, which resulted in some specific answers within each age category and these specific age-related findings do not apply to the entire population included in the hybrid care pathway.

Fourth, focus group interviews can in general suffer from social desirability bias which can impact the reliability of the collected data. However, we tried to limit this by making use of the predefined age groups.

Fifth, we choose to analyze the results from the perspective of the complete group rather than the individual perspectives within the groups with audio transcripts. The use of video transcripts would have also allowed the interpretation of individual nonverbal agree- or disagreements within the focus groups. Hereby a range of perspectives and agreements could have been provided which could have strengthened the quality of the final focus group results. To partly overcome this, however, we allowed each individual participant to make final remarks during the focus groups as described in the interview guide, which attributed to the overall intragroup agreements.

Finally, the evaluation we conducted was based on experiences with the hybrid care pathway of the Maasstad Hospital, and this also influences generalizability across other hybrid care pathways for hypertension.

## Conclusion

Based on this qualitative study, digital communication tools, telemonitoring applications, and digital lifestyle interventions that are part of hybrid care pathways for chronic diseases like hypertension should be offered as personalized as possible and should include personal interactions with healthcare providers. Tailoring the hybrid care pathway to various age groups can be a first step in this approach. This optimization could improve patients’ disease insight and adherence, which could ultimately lead to improved clinical outcomes.

## Supplemental Material

sj-docx-1-jpx-10.1177_23743735241297626 - Supplemental material for Patient Engagement in a Hybrid Care Pathway for Hypertension: Not One Size Fits All Supplemental material, sj-docx-1-jpx-10.1177_23743735241297626 for Patient Engagement in a Hybrid Care Pathway for Hypertension: Not One Size Fits All by Job van Steenkiste, Iris Verberk-Jonkers, Stéphanie de Koning, Joyce Voss-de Haan, Bianca de Jong-Verhagen and Daan Dohmen in Journal of Patient Experience

sj-docx-2-jpx-10.1177_23743735241297626 - Supplemental material for Patient Engagement in a Hybrid Care Pathway for Hypertension: Not One Size Fits All Supplemental material, sj-docx-2-jpx-10.1177_23743735241297626 for Patient Engagement in a Hybrid Care Pathway for Hypertension: Not One Size Fits All by Job van Steenkiste, Iris Verberk-Jonkers, Stéphanie de Koning, Joyce Voss-de Haan, Bianca de Jong-Verhagen and Daan Dohmen in Journal of Patient Experience

sj-docx-3-jpx-10.1177_23743735241297626 - Supplemental material for Patient Engagement in a Hybrid Care Pathway for Hypertension: Not One Size Fits All Supplemental material, sj-docx-3-jpx-10.1177_23743735241297626 for Patient Engagement in a Hybrid Care Pathway for Hypertension: Not One Size Fits All by Job van Steenkiste, Iris Verberk-Jonkers, Stéphanie de Koning, Joyce Voss-de Haan, Bianca de Jong-Verhagen and Daan Dohmen in Journal of Patient Experience
