# Supplementary material for: Patient Engagement in a Hybrid Care Pathway for Hypertension: Not One Size Fits All
Source: J Patient Exp. 2024 Dec 8;11:23743735241297626. doi: 10.1177/23743735241297626 (PMC11626661; doi:10.1177/23743735241297626)

**Supplementary file 1**: Included information files and education modules and screenshots from the Luscii app. The screenshots include material from the educational lessons on ‘lifestyle and blood pressure’ and ‘what is hypertension’?

| Self-care documents | Education modules |
| --- | --- |
| 1. What is home blood pressure monitoring? | 1. How does home blood pressure monitoring work? |
| 2. What should I do in case of a heat wave or a fever? | 2. How to correctly measure your blood pressure at home |
| 3. What should I do when vomiting or having diarrhoea? | 3. What is hypertension? |
| 4. I’m going for a holiday, how can I temporarily suspend my measurments | 4. Hypertension and lifestyle, a healthy diet, salt intake, physical activity, a healthy weight, smoking cessation, licorice |
| 5. What should I do when I deviated from my measurement schedule? | 5. Antihypertensive drugs |
| 6. What should I do when I forgot to take my antihypertensive drugs? | 6. Stress and hypertension |
| 7. I’m having symptoms that could be related to my blood pressure, what should I do? |  |
| 8. Can I still enter my blood pressure measurements from yesterday? |  |
| 9. How can I manually enter additional blood pressure measurements? |  |


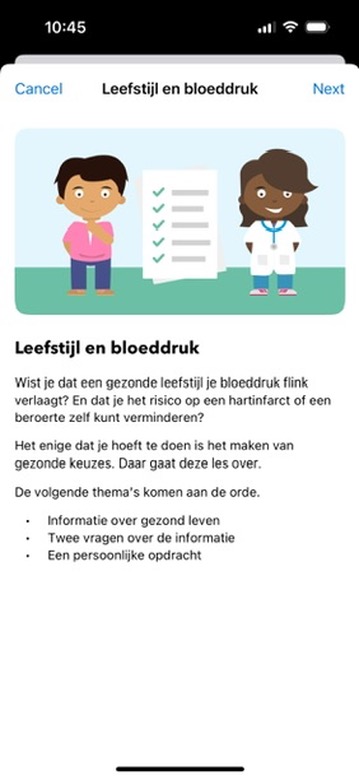

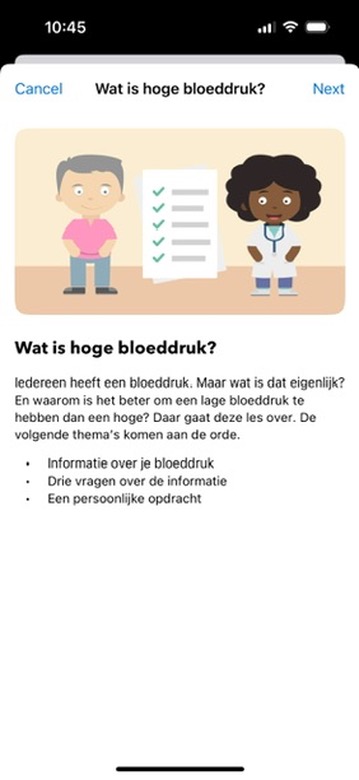

Supplement: sj-docx-1-jpx-10.1177_23743735241297626 - Supplemental material for Patient Engagement in a Hybrid Care Pathway for Hypertension: Not One Size Fits All [file sj-docx-1-jpx-10.1177_23743735241297626.docx]
