# Supplementary material for: Patient Engagement in a Hybrid Care Pathway for Hypertension: Not One Size Fits All
Source: J Patient Exp. 2024 Dec 8;11:23743735241297626. doi: 10.1177/23743735241297626 (PMC11626661; doi:10.1177/23743735241297626)
Supplement: sj-docx-2-jpx-10.1177_23743735241297626 - Supplemental material for Patient Engagement in a Hybrid Care Pathway for Hypertension: Not One Size Fits All [file sj-docx-2-jpx-10.1177_23743735241297626.docx]

**Supplementary file 2: interview guide**

**Total time for each interview: 2 hours**

**1. Introduction (20 minutes)**

- Welcome and introduction of participants from Maasstad
- Explanation of research purposes and explanation of the hybrid care pathway
- Guarantee of anonymity + explanation of audio recording
- Request phones off/silent
- Tutoring, completing name tags
- Emphasize that there are no wrong answers
- Explanation of the duration of the focus group
- Explanation of the surveys
- Explanation of the timekeeper role (Bart), in order to be able to discuss all topics sufficiently, we sometimes have to choose to end a discussion
- Announce start of the interview by asking: who are you and why did you choose home monitoring?

**2. How to live with hypertension in a healthy way (30 minutes)**

Every one of you has high blood pressure. From the moment you know you have high blood pressure; everyone tries to live with it as best as possible in their own way. We would like to know how you do that. Think about the two following questions for yourself for a few minutes and write what you are thinking about on post-its. Grab a different post it for each topic.

- What might you have done differently from the moment you knew you had high blood pressure?
- What is important to you to live as healthy as possible with high blood pressure**?**
- What information do you use?
- What do you think is a good way to help people with high blood pressure to live as healthy (in terms of lifestyle) as possible?

1. **Guidance from the care pathway and the use of the app (30 minutes)**

*You are being guided by the Maasstad Hospital as part of the hybrid carepathway. Also, you are using the home blood pressure monitoring app. Please answer the following questions:*

- What helped you in terms of achieving your lifestyle goals?
- What went well in terms of the app and the guidance provided?
- What can be improved?
- What did you miss?

1. **Supporting a healthy lifestyle (20 minutes)**

*In your opinion, what is a good method to provide lifestyle support?*

- General remarks? Now focus on the app
- How did you experience the messages you received in the app? And the communication in the care pathway, does this support you?

1. **Future (10 minutes)**

*What do you need in order to achieve and maintain your lifestyle goals and a healthy blood pressure in the future? What do you need from the hybrid care pathway?*

1. **Final remarks (10 minutes)**

- Questions, suggestions?
