# Supplementary material for: Patient Engagement in a Hybrid Care Pathway for Hypertension: Not One Size Fits All
Source: J Patient Exp. 2024 Dec 8;11:23743735241297626. doi: 10.1177/23743735241297626 (PMC11626661; doi:10.1177/23743735241297626)
Supplement: sj-docx-3-jpx-10.1177_23743735241297626 - Supplemental material for Patient Engagement in a Hybrid Care Pathway for Hypertension: Not One Size Fits All [file sj-docx-3-jpx-10.1177_23743735241297626.docx]

**Supplementary file 3: recruitment and inclusion flow diagram**
